# Predictors of rapid progression of estimated glomerular filtration rate among persons living with diabetes and/or hypertension in Ghana: Findings from a multicentre study

**DOI:** 10.1111/jch.14568

**Published:** 2022-09-06

**Authors:** Emmanuel Ofori, Kwadwo Faka Gyan, Solomon Gyabaah, Samuel Blay Nguah, Fred Stephen Sarfo

**Affiliations:** ^1^ Department of Medicine Komfo Anokye Teaching Hospital Kumasi Ghana; ^2^ Department of Medicine Kwame Nkrumah University of Science & Technology Kumasi Ghana

**Keywords:** estimated glomerular filtration rate, hypertension, multicenter study, predictors, rapid progression

## Abstract

In Ghana, the prevalence of chronic kidney disease (CKD) is 28.5% in diabetic hypertensive patients, 26.3% in hypertensives, and 16.1% in those with diabetes only. Trajectories of estimated glomerular filtration rate (eGFR) among patients with hypertension and diabetes are important for monitoring and instituting prompt interventions to prevent the development of CKD, especially in the face of limited access to renal replacement therapy. In this prospective multi‐center study conducted at five hospitals in Ghana, we assessed predictors of rapid eGFR progression among adults with hypertension and/or diabetes. Serum creatinine at baseline and 18 months were taken and eGFR determined using the CKD‐EPI formula. eGFR trajectory was defined as fast when the decline of GFR was ≥ 5 ml/min/1.73 m^2^ per year. A multivariable logistic regression model was fitted to identify predictors of the fast progression of eGFR. Total 13% of 1261 participants met the criteria for rapid decline in eGFR. The adjusted odds ratio, aOR (95%CI), of four factors adversely associated with fast progression of eGFR were: increasing age 1.20 (1.03–1.14), partial health insurance coverage for medications 1.48 (1.05–2.08), history of smoking 1.91 (1.11–3.27), angiotensin‐receptor blockade use 1.55 (1.06–2.25) while metformin use was protective .56 (.35–.90). Proportion with eGFR <60 ml/min increased from 14% at baseline to 19% at month 18. Effective health insurance policies to improve medication access and avoidance of smoking are interventions that may mitigate the rising burden of CKD in individuals with diabetes mellitus and/or hypertension.

## 1 INTRODUCTION

Chronic kidney disease (CKD) is a major public health challenge that contributes significantly to global morbidity and mortality from non‐communicable diseases. The rising burden of CKD is a major drawback to the United Nations target of reducing non‐communicable disease by a third by 2030.^1^ The major public health threat of CKD is due to increasing risk factors such as hypertension and diabetes.^2^ The global prevalence of CKD is between 11% and 13% of the world's population.^3^ In Africa, the prevalence of CKD is estimated to be 15.8% among the general population increasing to 34.5% in hypertension and 24.7% in diabetes subpopulations in Africa.^4^, ^5^ In Sub‐Saharan Africa, the prevalence of CKD is found to be nearly twice higher in high‐risk populations with comorbidities than in general populations.^5^ The prevalence of CKD in Ghana is 13.3% according to a recent study.^6^ Hypertension and diabetes are the leading risk factors for CKD worldwide.^7^ In a multi‐centre study conducted in Ghana, the prevalence of CKD was 28.5% in diabetic hypertensive patients, 26.3% in hypertensives, and 16.1% in those with diabetes only.^2^ CKD accentuates cardiovascular mortality approximately eight to tenfold in patients with diabetes and hypertension and is an important contributor to cardiovascular morbidity.^8^ In a prospective hospital‐based cohort of Ghanaians with hypertension and diabetes mellitus, CKD was found to be independently associated with stroke occurrence in a dose‐dependent manner.^9^

Renal function trajectory is defined as the change in estimated glomerular filtration rate (eGFR), in ml/min/1.73m2, over time and it is an indicator used in assessing the progression of CKD.^10^ The trajectory of eGFR has been studied in different populations with different characteristics. The trajectory of eGFR has been shown to be linear in non‐diabetics with a linear decline in renal function.^11^ The eGFR of those diagnosed with diabetes declines almost twice as rapidly as those without diabetes. Steeper declines were seen in diabetics who were either Black, had poor glycemic control or had other comorbidities such as hypertension.^12^ In another study of people with type 2 diabetes, those with younger age‐of‐onset or longer duration of type 2 diabetes had a more rapid decline in eGFR compared to those diagnosed in middle age or those with shorter duration of diabetes.^13^ This suggest that early and careful monitoring of eGFR changes in those with younger‐onset type 2 diabetes is essential as they are at the highest long‐term risk for kidney complications.

In Africa, there is a dearth of literature on the trajectory of eGFR among high‐risk patients. Such data among people with non‐communicable diseases are particularly important for monitoring and instituting prompt interventions to prevent the development of CKD, especially in the face of limited access to renal replacement therapy and the high cost involved in its management.^12^ The lack of reliable data in this area impedes the achievement of the sustainable development goal (SDG) 3.4 target of reducing by third, premature mortality from chronic NCDs through prevention and treatment by 2030. Thus, this study firstly sought to describe patterns of eGFR trajectory over 18 months and secondly to provide information on the factors associated with rapid decline in eGFR among patients with diabetes and hypertension using data from five hospitals in Ghana.

## 2 METHODS

### 2.1 Study design

The Ghana Access and Affordability Program (GAAP) was a prospective study involving adults with hypertension only, type 2 diabetes mellitus only, and those with both hypertension and type 2 diabetes mellitus. The health facilities included in the study were Komfo Anokye Teaching Hospital, Tamale Teaching Hospital, Agogo Presbyterian Hospital, Atua Government Hospital, and the Kings Medical center. Ethical approval was obtained from all study sites before the commencement of the study. The study protocol has been published.^14^

### 2.2 Eligibility criteria

The inclusion criteria were adults (i.e., aged 18 years and above) with a known diagnosis of hypertension and or type 2 diabetes mellitus. Hypertension was defined as a persistently elevated systolic blood pressure ≥140 mmHg and/or diastolic blood pressure ≥90 mmHg in patients who were on antihypertensive drugs at the enrollment visit. Diabetes mellitus was defined as a fasting blood glucose ≥7 mmol/L on two or more occasions, random blood glucose ≥11.1 mmol/L with symptoms, hemoglobin A1C (HbA1C) of > 6.5%, and on medications for the treatment of type 2 diabetes mellitus. Exclusion criteria included individuals with hypertensive urgencies or emergencies, hyperglycaemic or hypoglycaemic emergencies, and type 1 diabetes mellitus.

### 2.3 Evaluation of study participants

All eligible respondents provided informed consent before enrollment into the study. With the aid of trained research assistants, data comprising demographic information (i.e., age, sex, educational and employment status) and lifestyle behaviors (i.e., alcohol use, cigarette smoking, level of physical activities, and dietary behaviour) were collected through interviews and responses using questionnaires. Individuals were classified as physically active if they were regularly involved in moderate exercise or strenuous exercise for 4 or more hours per week. Alcohol use was categorized into current users (users of any form of alcoholic drinks) or never/former drinkers, while alcohol intake was categorized as low drinkers (1‐2 drinks per day for females and 1‐3 drinks per day for males) and high drinkers (>2 drinks per day for female and >3 drinks per day for male. One drink or 1 unit of alcohol = 8 g of alcohol. Smoking status was categorised into a current smoker (individuals who smoked any tobacco in the past 12 months) or a never or former smoker.^15^ Vegetable and fruit intake were assessed based on the number of daily servings per week. Further information collected included the duration of hypertension or diabetes diagnosis and current medications lists. Study nurses collected anthropometric data from participants and the Body Mass Index (BMI) of each participant was then derived by dividing the weight in kilograms by the square of the height in meters.

### 2.4 Laboratory measurements

An International Organization for Standardization (ISO)‐certified and quality‐assured laboratory was contracted to run all biochemical panels which included serum creatinine, and hemoglobin A1C for participants with diabetes. Samples were transported to the laboratory by trained phlebotomists on the same day of collection often within 4 h or where not feasible (Atua Government Hospital and Kings Medical center), samples were stored in a freezer before being transported to the laboratory the next day. The eGFR was determined from serum creatinine using the CKD‐EPI formula.^14^ CKD was defined as either eGFR < 60 ml/min/1.73 m^2^ with or without trace proteinuria and above or eGFR ≥60 ml/min with proteinuria according to the Kidney Disease Improving Global Outcome.^15^ Serum creatinine at baseline and the end of the study at 18 months were taken.

### 2.5 Primary outcome

Rapid decline of eGFR defined as more than 5 ml/min/1.73m^2^ per year.^16^

### 2.6 Secondary outcome

The frequency or proportion of study participants according to categories of eGFR trajectory into five patterns, namely, rapid decline, slow decline, stable, slow improvement, rapid improvement. This was based on the following definitions:

- Rapid decline =  eGFR decline ≤ –5 ml/min/1.73 m^2^ per year
- steady decline = eGFR decline up from 0 to –4.9 ml/min/1.73 m^2^ per year
- stable or no change = change in eGFR = 0 ml/min/1.73 m^2^ per year
- steady improvement = increased eGFR from 0 to 4.9 ml/min/1.73 m^2^ per year
- rapid improvement = increased eGFR by ≥5 ml/min/1.73 m^2^ per year

### 2.7 Statistical analysis

Baseline characteristics of participants with rapid versus normal eGFR progression were compared using the student's *t*‐test for means and proportions were compared using chi‐squared tests or Fisher's exact test for proportions with subgroupings with counts less than five. A multivariable logistic regression model was fitted to identify factors associated with rapid progression of eGFR. Independent variables evaluated included age, sex, location of residence, employment status, health insurance cover (as to whether all medications were covered fully or partially with out‐of‐pocket payments), previous cigarette smoking, current alcohol use, physical activity, table added salt, fruit and vegetable intake, level of health care institution (primary, secondary, or tertiary), central obesity, duration of hypertension or diabetes, number and classes of antihypertensive and anti‐glycemic medications, and baseline systolic and diastolic BP as well as baseline HBA1c. Variable selection was based on clinical and empirical significance of covariates in the model. Variables were included in the multivariate analyses upon meeting a *p*‐value cutoff of < .05 in bivariate unadjusted regression analysis. In all analyses, two‐tailed *p* < .05 were considered statistically significant. Statistical analysis was performed using R Statistical software Ver 4.1.1.

## 3 RESULTS

### 3.1 Baseline characteristics of study participants

The study included 1261 participants from the 5 study sites. The mean (SD) of study participants was 57.5 (11.6) years with a female preponderance 79.5%. Of the study participants, 45.7% resided in rural dwellings, 23.9% in peri‐urban, and 30.4% in urban settings. There were 680 (53.9%) with hypertension only, 425 (33.7%) with hypertension and diabetes and 156 (12.4%) with diabetes only.

### 3.2 Mean eGFR changes from baseline to month 18

There was an overall decline in the mean eGFR from 77.22 ml/min/1.73 m2 at baseline to 73.84 ml/min/1.73 m2 at month 18, *p* < .0001. By sub‐groups, mean eGFR at baseline vs month 18 were 76. 59 versus 73.56 ml/min/1.73 m2 (*p* = .0003, 95% CI (1.41–4.65) for hypertension only group; 83.56 versus 80.63 ml/min/1.73 m2 (*p* = .03 95%CI (.33–5.52) for diabetes only group; and 75.90 versus 71.79 ml/min/1.73m2 (*p* = .0007, 95% CI (1.73–6.50)]. However, for participants with CKD at baseline, that is, eGFR ˂ 60 ml/min/1.73m2, there was no significant change in the mean eGFR from a baseline of 46.73 ml/min/1.73 m2 to 46.44 ml/min/1.73 m2 at the end of follow up (*p* = .95, 95%CI (–2.47 to 2.62)).

### 3.3 Trajectory of eGFR patterns over 18 months

Of the study participants, 13% had a rapid decline in eGFR, 10% had a steady decline, 30% had stable eGFR or no change in eGFR, 16% had steady improvement, and 31% had rapid improvement in the eGFR over 18 months. Furthermore, while at baseline 42% of participants had eGFR > = 89 ml/min, at month 18 the proportion at this eGFR dropped to 32%. However, those with eGFR 60–89 ml/min at baseline increased from 44% to 48%, eGFR 30–59 ml/min increased from 9% to 14%, eGFR 15–29 ml/min increased from 1% to 4% with no changes in eGFR < 15 ml/min (0% vs. 0%). (Table 1) Overall, the proportion with eGFR < 60 ml/min increased from 14% at baseline to 19% at month 18.

**TABLE 1:** Comparison of proportion of Ghanaian study participants by eGFR categories at baseline and month 18

eGFR categories	Baseline	Month 18	*p*‐value
>89 ml/min	529 (42.0)	403 (32.0)	<.0001
60‐89 ml/min	555 (44.0)	606 (48.0)	
45‐59 ml/min	117 (9.0)	180 (14.0)	
30–44 ml/min	45 (4.0)	50 (4.0)	
15–29 ml/min	13 (1.0)	18 (1.0)	
<15 ml/min	2 (.0)	4 (.0)	

### 3.4 Comparison of clinical and demographic characteristics of study participants by progression of eGFR

There were 164 (13.0%) with rapid progression of eGFR. The mean age in years of participants with rapid eGFR progression was higher compared to those with normal progression [60.3 ± 10.5 vs. 57.1 ± 11.8; (*p* = .001)] Table 2. For participants whose medications were fully paid for by the National Health Insurance Scheme, a significantly lower proportion had fast progression of eGFR compared with those whose medications were partially paid [67 (40.9%) vs. 97(59.1%); *p* = .018]. A higher proportion of participants with hypertension only, as compared with those with diabetes mellitus only and with both diabetes mellitus and hypertension had faster progression of eGFR (*p* = .032). In addition, those with a history of smoking were more likely to progress faster compared to those who do not (*p* = .007). The progression of eGFR was rapid among those with a history of stroke (*p* = .031), use of angiotensin receptor blockers (ARB) (*p* = .006), use of diuretics (*p* = .013), lower number of antidiabetics being used (*p* = .023), non‐use of metformin (*p* = .024), and higher number of antihypertensives (*p* = .017). The baseline characteristics of those with hypertension only, diabetes mellitus only and hypertension with diabetes are shown in Table S1.

**TABLE 2:** Comparison of demographic & clinical features of rapid and non‐rapid progression of eGFR in all patients

	GFR progression			
Characteristic	Normal	Rapid	Total	*p*‐value
Age in years, *mean (SD)*	57.1 (11.8)	60.3 (10.5)	57.5 (11.6)	.001
Male sex, *n (%)*	218 (19.9)	40 (24.4)	258 (20.5)	.181
Residence				.478
Rural	503 (45.9)	73 (44.5)	576 (45.7)	
Periurban	267 (24.3)	35 (21.3)	302 (23.9)	
Urban	327 (29.8)	56 (34.1)	383 (30.4)	
Educational level				.360
None	359 (32.7)	60 (36.6)	419 (33.2)	
Primary	221 (20.1)	34 (20.7)	255 (20.2)	
Secondary	396 (36.1)	59 (36)	455 (36.1)	
Tertiary	121 (11)	11 (6.7)	132 (10.5)	
Employment				.744
Employed	763 (69.6)	112 (68.3)	875 (69.4)	
Unemployed	334 (30.4)	52 (31.7)	386 (30.6)	
Income				.127
>GH⊄1000	57 (5.2)	8 (4.9)	65 (5.2)	
GH⊄ 210–1000	353 (32.2)	38 (23.2)	391 (31)	
<GH⊄ 210	360 (32.8)	62 (37.8)	422 (33.5)	
Unknown	327 (29.8)	56 (34.1)	383 (30.4)	
Drugs paid with NHIS *n* (%)				.018
Fully paid	557 (50.8)	67 (40.9)	624 (49.5)	
Partially paid	540 (49.2)	97 (59.1)	637 (50.5)	
Disease class				.032
Hypertension	578 (52.7)	102 (62.2)	680 (53.9)	
Diabetes Mellitus	144 (13.1)	12 (7.3)	156 (12.4)	
Hypertension + Diabetes Mellitus	375 (34.2)	50 (30.5)	425 (33.7)	
Ever smoked, *n (%)*	70 (6.4)	20 (12.2)	90 (7.1)	.007
Alcohol intake, *n (%)*	95 (8.7)	18 (11)	113 (9)	.333
Salt added to food, *n (%)*	158 (14.4)	25 (15.2)	183 (14.5)	.775
Physical activity, *n (%)*	367 (33.5)	57 (34.8)	424 (33.6)	.742
Weekly hours exercising, *mean (SD)*	22.7 (26)	23.4 (31.5)	22.7 (26.7)	.751
Days of Fruit per week, *mean (SD)*	2.6 (2.1)	2.4 (1.8)	2.6 (2.1)	.122
Daily Fruit servings, *mean (SD)*	1.7 (1.6)	1.8 (2)	1.7 (1.7)	.543
Days/week vegetables, *mean (SD)*	5.5 (1.9)	5.6 (1.8)	5.5 (1.9)	.531
Daily vegetable servings, mean *(SD)*	2.2 (1.5)	2.3 (1.5)	2.2 (1.5)	.624
Heart failure *n (%)*	75 (6.8)	15 (9.1)	90 (7.1)	.284
Stroke *n (%)*	55 (5)	15 (9.1)	70 (5.6)	.031
BMI *mean (SD)*	26.6 (5.4)	26 (5.8)	26.5 (5.5)	.204
Waist Circumference, *mean (SD)*	95.4 (13.6)	94.1 (12.7)	95.2 (13.5)	.267
Duration of hypertension in years hypertensive, *mean (SD)*	7.5 (6.8)	8.4 (7.2)	7.7 (6.9)	.159
Duration of DM (years), *mean (SD)*	4.1 (6.1)	4.2 (8.1)	4.1 (6.4)	.838
Baseline Systolic BP mean (SD)	139.5 (20.7)	138.2 (18.3)	139.3 (20.4)	.456
Baseline HBA1C, mean (SD)	8.5 (2.4)	8.7 (3.5)	8.5 (2.5)	.519
Baseline eGFR, mean (SD)	79.4 (14.3)	62.5 (13.8)	77.2 (15.3)	<.001
*Baseline eGFR categories*				<.001
>89 ml/min	529 (48.2)	0 (0)	529 (42)	
60–89 ml/min	447 (40.7)	108 (65.9)	555 (44)	
45–59 ml/min	79 (7.0)	38 (23.0)	117 (9.0)	
30–44 ml/min	31 (3.0)	14 (9.0)	45 (4.0)	
15–29 ml/min	10 (1.0)	3 (2.0)	13 (1.0)	
<15 ml/min	1 (.0)	1 (1.0)	2 (.0)	
ACE‐Inhibitors use, *n (%)*	452 (41.2)	59 (36)	511 (40.5)	.203
ARB use, *n (%)*	288 (26.3)	60 (36.6)	348 (27.6)	.006
Beta‐Blockers use, *n (%)*	91 (8.3)	18 (11)	109 (8.6)	.255
Calcium Channel Blockers, *n (%)*	721 (65.7)	115 (70.1)	836 (66.3)	.267
Diuretics, *n* (%)	348 (31.7)	68 (41.5)	416 (33)	.013
Methyldopa, *n (%)*	159 (14.5)	21 (12.8)	180 (14.3)	.564
Hydralazine, *n (%)*	10 (.9)	1 (.6)	11 (.9)	.698
No. of antidiabetics, *mean (SD)*	1.1 (1.2)	.8 (1.2)	1 (1.2)	.023
Metformin, *n (%)*	491 (44.8)	58 (35.4)	549 (43.5)	.024
Sulphonylurea, *n* (%)	346 (31.5)	43 (26.2)	389 (30.8)	.169
Thiazolidinedione, *n* (%)	196 (17.9)	22 (13.4)	218 (17.3)	.160
Insulin, *n* (%)	129 (11.8)	12 (7.3)	141 (11.2)	.092
DPP4, *n* (%)	3 (.3)	1 (.6)	4 (.3)	.475
Statin, *n* (%)	101 (9.2)	14 (8.5)	115 (9.1)	.781
Antiplatelet, *n* (%)	114 (10.4)	16 (9.8)	130 (10.3)	.803
No. of Antihypertensive *mean(SD)*	1.9 (1)	2.1 (.9)	1.9 (1)	.017
Hillbone score, *mean (SD)*	18.4 (3.4)	18 (3.0)	18.3 (3.4)	.159

Abbreviations: ACE, angiotensin converting enzyme; ARB, angiotensin receptor blocker, BMI, body mass index; DD4 = Dipeptidyl peptidase‐4; DM, diabetes mellitus; NHIS, National Health Insurance Scheme.

*p*‐value < .005 is significant. Table 2 indicates the clinical and demographic characteristics of all patients in the study. Advanced Age, drug paid by NHIS, disease class, history of smoking, ARB use, number of antidiabetic medications, metformin use are the significant characteristics that affect the patient's eGFR.

### 3.5 Factors associated with rapid eGFR progression

Five (5) factors were independently associated with rapid progression in the entire study population. The adjusted odds ratio (aOR) with 95%CI for these factors include: increasing age [aOR of 1.2(1.03,1.14), *p* = .017] each 10 years older, partial NHIS coverage for medications, [aOR of 1.48(1.05,2.08), *p* = .024], history of having ever smoked cigarette [aOR of 1.91(1.11,3.27), *p* = .019], and ARB use, [aOR of 1.55(1.06,2.25), *p* = .022]. For those on metformin, they were less likely to have fast progression of eGFR, [aOR of .56(.35,.9), *p* = .016]. For those with stroke, crude analysis showed the odds of fast progression in eGFR was increased 91% but lost its statistical significance on adjusted analysis at 72%, [aOR of 1.72% (.92,3.19), *p* = .088] as shown in Table 3.

**TABLE 3:** Determinant of Rapid eGFR progression in all patients

	Crude analysis		Adjusted analysis	
Characteristic	OR (95%CI)	*p*‐value	AOR (95%CI)	*p*‐value
Age in years, *per 10 years*	1.27 (1.1,1.47)	.001	1.2 (1.03,1.4)	.017
Male sex	1.3 (.88,1.91)	.182		
Residence				
Rural	Ref			
Periurban	.9 (.59,1.39)	.642		
Urban	1.18 (.81,1.72)	.387		
Educational level				
None	Ref			
Primary	.92 (.59,1.45)	.720		
Secondary	.89 (.61,1.31)	.560		
Tertiary	.54 (.28,1.07)	.077		
Unemployed	1.06 (.75,1.51)	.744		
Income				
>GH⊄1000	Ref			
GH⊄ 210–1000	.77 (.34,1.73)	.522		
<GH⊄ 210	1.23 (.56,2.7)	.611		
Unknown	1.22 (.55,2.7)	.623		
Partial NHIS drugs payment	1.49 (1.07,2.08)	.018	1.48 (1.05,2.08)	.024
Ever Smoked	2.04 (1.2,3.45)	.008	1.91 (1.11,3.27)	.019
Alcohol intake	1.3 (.76,2.22)	.334		
Salt added to food	1.07 (.68,1.69)	.776		
Physical activity	1.06 (.75,1.5)	.742		
Weekly hours exercising	1.00 (.99,1.01)	.751		
Days of Fruit per week	.94 (.86,1.02)	.122		
Daily Fruit servings	1.03 (.94,1.13)	.543		
Days/week vegetables	1.03 (.94,1.12)	.531		
Daily vegetable servings	1.03 (.92,1.14)	.624		
Heart failure	1.37 (.77,2.45)	.286		
Stroke	1.91 (1.05,3.46)	.034	1.72 (.92,3.19)	.088
Body Mass Index	.98 (.95,1.01)	.204		
Waist Circumference	.99 (.98,1.00)	.267		
Hypertension duration (yrs)	1.02 (.99,1.04)	.160		
Duration of DM (years)	1.00 (.98,1.03)	.838		
ACE‐Inhibitors use	.8 (.57,1.13)	.204		
ARB use	1.62 (1.15,2.29)	.006	1.55 (1.06,2.25)	.022
Beta‐Blockers use	1.36 (.8,2.33)	.256		
Calcium Channel Blockers	1.22 (.86,1.75)	.267		
Diuretics	1.52 (1.09,2.13)	.014	1.32 (.88,1.97)	.174
Methyldopa	.87 (.53,1.41)	.564		
Hydralazine	.67 (.08,5.24)	.700		
No. of antidiabetics	.85 (.73,.98)	.024	.96 (.78,1.2)	.745
Metformin	.68 (.48,.95)	.024	.56 (.35,.9)	.016
Sulphonylurea	.77 (.53,1.12)	.170		
Thiazolidinedione	.71 (.44,1.15)	.161		
Insulin	.59 (.32,1.1)	.096		
DPP4 Inhibitor	2.24 (.23,21.64)	.487		
Statin	.92 (.51,1.65)	.781		
Antiplatelet	.93 (.54,1.62)	.803		
Number of antihypertensive	1.22 (1.04,1.44)	.018	.96 (.78,1.2)	.745
Hillbone score	.96 (.91,1.02)	.159		

Abbreviations: ACE, angiotensin converting enzyme; DARB, Angiotensin Receptor Blocker; DD4, Dipeptidyl peptidase‐4; DM, diabetes mellitus; NHIS, National Health Insurance Scheme.

*p*‐value < .005 is significant. In Table 3, the determinants of rapid progression in eGFR reported for all study participants as adjusted odds ratio (aOR) with 95%CI include increasing age per 10 years, partial NHIS drugs payment, ever smoked before and ARB use. Patients on metformin were less likely to have fast progression of eGFR.

### 3.6 Stratified analyses

On subgroup analysis of individuals living with diabetes mellitus only, salt added to food at table was independently predictive of rapid eGFR decline, aOR of 4.19 (95%CI: 1.22–14.40) shown in Table 4. Furthermore, among those with both hypertensin and diabetes, three factors independently associated with rapid decline in eGFR were partial insurance coverage of medications, smoking history and duration of diabetes diagnosis (Table 5). None of the factors in those with hypertensives only was independently associated with rapid progression (Table S2).

**TABLE 4:** Factors associated with Rapid eGFR progression in patients with diabetes mellitus only

	Crude analysis		Adjusted analysis	
Characteristic	OR (95%CI)	*p*‐value	AOR (95%CI)	*p*‐value
Age in years, *per 10 years*	1.6 (.92,2.78)	.094		
Male sex	.79 (.16,3.82)	.773		
Residence				
Rural	1			
Periurban	4.31 (.49,38.33)	.190		
Urban	5.39 (.62,46.52)	.126		
Educational level				
None	1			
Primary	.68 (.12,3.98)	.672		
Secondary	1.02 (.27,3.86)	.971		
Tertiary	0 (0,Inf)	.993		
Unemployed	.83 (.17,4)	.815		
Income				
> GH⊄1000	1			
GH⊄ 210–1000	.5 (.08,2.95)	.444		
<GH⊄ 210	.13 (.01,1.52)	.103		
Unknown	.45 (.07,2.84)	.399		
Partial NHIS drugs payment	1.22 (.37,3.95)	.746		
Ever smoked	0 (0, Inf)	.990		
Alcohol intake	1.22 (.14,10.41)	.857		
Salt added to food	4.33 (1.3,14.48)	.017	4.19 (1.22,14.4)	.023
Physical activity	.56 (.12,2.66)	.464		
Weekly hours exercising	.99 (.96,1.01)	.353		
Days of Fruit per week	.99 (.73,1.33)	.944		
Daily Fruit servings	.96 (.59,1.57)	.884		
Days/week vegetables	1.13 (.78,1.64)	.502		
Daily vegetable servings	1.27 (.93,1.74)	.127		
Heart failure	7.0 (1.14,42.97)	.036	6.49 (.96,43.87)	.055
Stroke	0 (0, Inf)	.994		
Body Mass Index	.93 (.81,1.07)	.299		
Waist Circumference	.99 (.94,1.04)	.668		
Duration of DM (years)	1.03 (.94,1.12)	.590		
ACE‐Inhibitors use	1.39 (.4,4.9)	.604		
ARB use	1.32 (.27,6.47)	.736		
Beta‐Blockers use	0 (0, Inf)	.993		
Diuretics	0 (0, Inf)	.993		
No. of antidiabetics	1.38 (.57,3.36)	.479		
Metformin	.91 (.11,7.71)	.931		
Sulphonylurea	1.69 (.49,5.87)	.407		
Thiazolidinedione	2 (.61,6.53)	.251		
Insulin	.32 (.07,1.53)	.155		
DPP4 Inhibitor	0 (0, Inf)	.993		
Statin	.64 (.08,5.23)	.674		
Antiplatelet	1.86 (.37,9.34)	.453		
Hillbone score	1.18 (.53,2.59)	.688		

Abbreviations: ACE, angiotension converting enzyme; ARB, Angiotension Receptor Blocker; BMI, body mass index; DM, diabetes mellitus; NHIS, National Health Insurance Scheme.

*p*‐value < .005 is significant. Table 4 indicates that On subgroup analysis of individuals living with diabetes mellitus partial insurance payment of the cost of medications by the NHIS was the only factor associated with rapid eGFR progression.

**TABLE 5:** Factors associated with Rapid eGFR progression in patients with both hypertension and diabetes

Characteristic	Crude analysis		Adjusted analysis	
	OR (95%CI)	*p*‐value	AOR (95%CI)	*p*‐value
Age in years, *per 10 years*	1.25 (.95,1.65)	.115		
Male sex	1.26 (.63,2.54)	.511		
Residence				
Rural	1			
Periurban	1.17 (.52,2.64)	.712		
Urban	1.27 (.61,2.65)	.520		
Educational level				
None	1			
Primary	1.07 (.5,2.28)	.863		
Secondary	.76 (.37,1.59)	.466		
Tertiary	.54 (.17,1.66)	.280		
Unemployed	1.82 (1.01,3.3)	.048		
Income				
>GH⊄1000	1			
GH⊄ 210–1000	.9 (.23,3.48)	.883		
<GH⊄ 210	1.81 (.5,6.59)	.366		
Unknown	1.71 (.48,6.1)	.411		
Partial NHIS drugs payment	2.11 (1.1,4.04)	.024	1.94 (1.00, 3.76)	.048
Ever smoked	2.83 (1.25,6.43)	.013	2.75 (1.19, 6.33)	.017
Alcohol intake	1.7 (.62,4.7)	.306		
Salt added to food	1.26 (.53,2.97)	.602		
Physical activity	1.05 (.57,1.94)	.882		
Weekly hours exercising	1.0019 (.99,1.014)	.751		
Days of Fruit per week	.91 (.78,1.06)	.215		
Daily fruit servings	1.01 (.82,1.25)	.899		
Days/week vegetables	.9 (.78,1.04)	.146		
Daily vegetable servings	1.07 (.92,1.24)	.358		
Heart failure	.79 (.23,2.7)	.708		
Stroke	1.87 (.78,4.52)	.163		
Body Mass Index	.9955 (.93,1.05)	.877		
Waist Circumference	.99 (.97,1.01)	.612		
Hypertension duration (yrs)	1.01 (.97,1.05)	.554		
Duration of DM (years)	1.05 (1.01,1.09)	.015	1.04 (1.01,1.08)	.023
ACE‐Inhibitors use	.9 (.5,1.63)	.736		
ARB use	1.3 (.71,2.38)	.393		
Beta‐Blockers use	1.54 (.51,4.71)	.446		
Calcium Channel Blockers	.6 (.33,1.09)	.097		
Diuretics	1.13 (.54,2.37)	.749		
Methyldopa	1.04 (.46,2.33)	.922		
Hydralazine	0 (0,Inf)	.988		
No. of antidiabetics	.97 (.65,1.45)	.885		
Metformin	1.22 (.42,3.59)	.716		
Sulphonylurea	1.08 (.57,2.06)	.804		
Thiazolidinedione	.76 (.41,1.43)	.401		
Insulin	.97 (.45,2.1)	.945		
DPP4 Inhibitors	3.81 (.34,42.75)	.279		
Statin	1.19 (.57,2.51)	.643		
Antiplatelet	.84 (.38,1.88)	.679		
No. of Antihypertensive	.93 (.66,1.31)	.670		
Hillbone score	.92 (.83,1.02)	.133		

Abbreviations: ACE, angiotension converting enzyme; ARB, Angiotension Receptor Blocker; BMI, body mass index; DD4, dipeptidyl peptidase‐4; DM, diabetes mellitus; NHIS, National Health Insurance Scheme.

*p*‐value < .005 is significant. Table 5 shows that none of the factors in those with hypertensives only was independently associated with rapid progression.

## 4 DISCUSSION

We found in this prospective multi‐centre Ghanaian study that 14% of individuals living with hypertension and or diabetes had CKD with eGFR < 60 ml/min increasing to 19% over 18 months of follow‐up. Furthermore, 13.0% of our study sample exhibited a rapid decline in eGFR of ≥5 ml/min/1.73 m^2^. A population‐based study in Norway among patients in CKD stage 3, reported 6% showing a decline in eGFR of ‐5 ml/min/1.73 m^2^ per year.^17^ In a retrospective study in the USA among non‐proteinuric hypertensive patients with and without diabetes, the fraction of patients with a decline in eGFR > 3.5 ml/min/year was 7.2% among patients with diabetes and 6.0% among those without diabetes.^18^ Among community‐dwelling older adults aged > 65years of Chinese ethnicity with or without co‐morbidities followed up for 10 years, 1.8% of them had eGFR decline ‐4 ml/min/1.73 m^2^ per year.^19^ Admittedly these cited studies involved a heterogeneous study sample and eGFR decline rates were different across the studies. We choose a less conservative cut‐off of ≥5 ml/min/1.73 m^2^ per year. Even so, we observed a significant burden of rapid declines in kidney function among Ghanaians living with hypertension and or diabetes compared with previously cited studies.^17^, ^18^, ^19^

In the present study, we found increasing age, partial health insurance coverage for medications, history of smoking, and prescription of ARB were associated with a rapid decline in eGFR while patients on Metformin had a slow progression. Each 10‐year rise in age was associated with 20% increased odds of faster progression of eGFR. Age is an important non‐modifiable risk factor that impacts the trajectory of eGFR. Both morphological and functional changes occur in the senescent kidney.^20^ Global glomerulosclerosis increases steadily with increasing age in the absence of any existing co‐morbidity such as hypertension or diabetes and there is a gradual decline in the overall number of nephrons as one age.^21^ Overall, the age‐related decline in eGFR usually begins after the third decade of life and most often progresses at a rate of 8 ml/min per 1.73 m^2^ per decade^22^, ^23^, ^24^ which usually accelerates after the age of 50–60 years.^25^ The progress of this process may be influenced by superimposed diseases such as diabetes and hypertension.^26^ Diabetes, hypertension and aging have been found as independent key predictors of CKD.^27^

As noted in a previous study, we observed a higher rate of progression of eGFR among individuals with partial insurance coverage for antihypertensive and or antidiabetic medications.^28^ Access to essential medicines to manage non‐communicable diseases is severely limited in public sector settings in Low‐ and Middle‐Income Countries and this adversely impact disease outcomes.^29^, ^30^ In this cohort, we have shown that the absence of National Health Insurance Scheme (NHIS) was adversely associated with poor glycemic control.^31^ Also uncontrolled hypertension was associated with poor adherence to antihypertensive therapy due to reported difficulty in obtaining medications in this cohort.^32^ We found that a history of cigarette smoking was associated with a higher adjusted odds of rapid eGFR progression by 91% compared with those who did not smoke. A meta‐analysis showed cigarette smoking, that is, ever smokers, current smokers, and former smokers as an independent risk factor for incident CKD.^33^ Although smoking is dose‐dependently related to incident kidney failure, prolonged cessation of smoking significantly reduced this risk.^34^, ^35^ Also salt intake was found to be significantly associated with rapid progression of eGFR in patients with diabetes. Salt added to food increases urinary albumin excretion which significantly increases risk of cardiovascular events and progression of diabetic nephropathy.^36^, ^37^, ^38^

An intriguing observation was that of a faster progression of eGFR among participants on ARBs. A possible explanation could be indication bias where ARBs were initiated by clinicians among patients who already have proteinuria to avert progressive renal function decline.^39^ On the other hand, in line with evidence from previous studies, metformin had a protective effect on the decline in eGFR.^39^, ^40^ CKD progression towards end‐stage kidney disease is through the loss of kidney cells and their replacement by extracellular matrix independently of the etiology of underlying primary disease.^40^ Evidence from various studies has shown that metformin prevents renal parenchymal loss by targeting the fibrotic pathways through a complex pathway of activating adenosine monophosphate activating protein kinase (AMPK) and mammalian target of rapamycin (mTOR) inhibition.^40^ Thus, through its antifibrotic effect metformin can reduce the rate of decline of renal function and prevent the progression of CKD.

### 4.1 Implications

Our study has implications for individuals living with hypertension and diabetes, health care providers, and policymakers/directors. At an individual patient level, adherence to therapeutic lifestyle interventions such as smoking cessation and medications prescribed by clinicians would improve blood pressure and glycemic control and avert decline in renal function. Given the observed association between increasing age and risk of rapid progression of eGFR, the ageing population living with diabetes or hypertension and other high CV risk groups^41^, ^42^, ^43^, ^44^ should be educated on the need for regular screening of renal function. For clinicians, more frequent assessment of eGFR among hypertensive and diabetics perhaps yearly may help with the identification of individuals at risk of rapid progression of eGFR, for referral to a nephrologist or a specialist. While difficult to explain the association of the use of ARBs and the rapid progression of CKD, those on ARBs may require more frequent monitoring. At the policy level, an expanded and affordable health insurance policy to cover all medications for patients with hypertension and or diabetes will undoubtedly improve access and affordability to these lifesaving medications to prevent end‐organ complications such as end‐stage kidney disease.

### 4.2 Limitations

This prospective cohort study had some important limitations. Estimated glomerular filtration rates were assessed at two‐time points, baseline and 18 months later and hence we could not evaluate fluctuations in eGFR in between these two‐time points. Also, there could be selection bias as only participants who had both baseline renal function and end of study renal function were included in this study. Despite these limitations, this present study fills an important gap in the literature by identifying predictors of rapid decline in renal function among individuals with diabetes and or hypertension in resource‐limited settings in Ghana.

## 5 CONCLUSION

The trajectory of eGFR is affected by both modifiable and non‐modifiable risk factors including age, coverage of health insurance, history of smoking, and the use of angiotensin receptor blockade (ARB) and metformin in individuals with diabetes mellitus and/or hypertension. Health sector policies should be targeted at expanding coverage and accessibility of diabetes and hypertension medications to improve outcomes.

## AUTHOR CONTRIBUTIONS

Conceptualization: Fred Stephen Sarfo. Data curation: Fred Stephen Sarfo. Formal analysis: Samuel Blay Nguah. Investigation: Fred Stephen Sarfo. Methodology: Fred Stephen Sarfo, Emmanuel Ofori, Kwadwo Faka Gyan, Solomon Gyabaah. Project administration: Fred Stephen Sarfo. Supervision: Fred Stephen Sarfo. Writing‐ original draft: Emmanuel Ofori, Kwadwo Faka Gyan, Solomon Gyabaah. Writing‐review & editing: Emmanuel Ofori, Kwadwo Faka Gyan, Solomon Gyabaah, Samuel Blay Nguah, Fred Stephen Sarfo.

## CONFLICT OF INTEREST

Authors declare no conflict of interest.

## CONSENT

Informed consent was obtained from participants

## Supporting information

Supporting information
