# Supplementary material for: Predictors of rapid progression of estimated glomerular filtration rate among persons living with diabetes and/or hypertension in Ghana: Findings from a multicentre study
Source: J Clin Hypertens (Greenwich). 2022 Sep 6;24(10):1358–69. doi: 10.1111/jch.14568 (PMC9581086; doi:10.1111/jch.14568)
Supplement: Supplementary file 1 — Supporting information [file JCH-24-1358-s001.docx]

**SUPPLEMENTARY INFORMATION**

TITLE: **Predictors of Rapid Progression of estimated Glomerular Filtration Rate Among Persons Living with Diabetes and/or Hypertension in Ghana: Findings from a multicentre study**

**Authors**:

Emmanuel Ofori^1^, Kwadwo Faka Gyan^1^, Solomon Gyabaah^1^, Samuel Blay Nguah^1,2^, Fred Stephen Sarfo^1,2^

**Corresponding Author:**

Prof. Fred Stephen Sarfo

Kwame Nkrumah University of Science & Technology,

Kumasi, Ghana

Tel: 233-243-448464

Email address: [Stephensarfo78@gmail.com](mailto:Stephensarfo78@gmail.com)

**Supplementary table 1. Baseline characteristics of those with hypertension only, diabetes mellitus only and hypertension with diabetes**.

| Variable | HYPERTENSION ONLY | DM ONLY | BOTH HPT AND DM | P-value |
| --- | --- | --- | --- | --- |
| Age in years, mean (SD) | 58.4 (11.4) | 48.9 (10.8) | 59.2 (11) | < 0.001 |
| Male sex | 140 (20.6) | 31 (19.9) | 87 (20.5) | 0.98 |
| Serum Creatinine *mean (SD)* | 78.8 (25.8) | 68.4 (20.2) | 78.5 (34.9) | < 0.001 |
| Baseline eGFR | 76.6 (14.7) | 83.6 (10.4) | 75.9 (17.2) | < 0.001 |
| Proteinuria | 0.1 (0.4) | 0.1 (0.2) | 0.2 (0.6) | 0.007 |
| ACE-Inhibitors use *n (%)* | 264 (38.8) | 42 (26.9) | 205 (48.2) | < 0.001 |
| ARB use *n (%)* | 180 (26.5) | 21 (13.5) | 147 (34.6) | < 0.001 |
| Statin, n (%) | 21 (3.1) | 19 (12.2) | 75 (17.6) | < 0.001 |
| Antiplatelet, n (%) | 37 (5.4) | 16 (10.3) | 77 (18.1) | < 0.001 |
| Hillbone score, *mean (SD)* | 18.4 (3.4) | 18.8 (3.2) | 18.2 (3.3) | 0.390 |

**Supplementary table 2. Factors associated with rate of eGFR progression in patients with Hypertension only**

| **Characteristic** | **Crude Analysis** | | **Adjusted Analysis** | |
| --- | --- | --- | --- | --- |
|  | **OR (95%CI)** | **p-value** | **AOR (95%CI)** | **p-value** |
| Age in years per 10years | 1.2 (1.0,1.45) | 0.054 | 1.2 (0.99,1.46) | 0.058 |
| Male sex | 1.39 (0.85,2.27) | 0.186 |  |  |
| Residence |  |  |  |  |
| 0 | Ref |  |  |  |
| 1 | 0.85 (0.47,1.54) | 0.591 |  |  |
| 2 | 1.36 (0.82,2.25) | 0.237 |  |  |
| Educational level |  |  |  |  |
| 0 | Ref |  |  |  |
| 1 | 0.89 (0.49,1.63) | 0.711 |  |  |
| 2 | 0.95 (0.58,1.54) | 0.825 |  |  |
| 3 | 0.62 (0.26,1.46) | 0.273 |  |  |
| Unemployed | 0.77 (0.47,1.26) | 0.298 |  |  |
| Income |  |  |  |  |
| 0 | Ref |  |  |  |
| 1 | 0.62 (0.17,2.31) | 0.478 |  |  |
| 2 | 1.00 (0.27,3.60) | 1.000 |  |  |
| 3 | 1.01 (0.28,3.70) | 0.985 |  |  |
| Partial NHIS drugs payment | 1.34 (0.88,2.05) | 0.170 |  |  |
| Ever Smoked | 2,00 (0.97,4.09) | 0.059 | 1.8 (0.86,3.76) | 0.120 |
| Alcohol intake | 1.11 (0.58,2.14) | 0.756 |  |  |
| Salt added to food | 0.74 (0.39,1.41) | 0.364 |  |  |
| Physical activity | 1.09 (0.70,1.69) | 0.693 |  |  |
| Weekly hours exercising | 1.00 (0.99,1.01) | 0.382 |  |  |
| Days of Fruit per week | 0.95 (0.85,1.06) | 0.340 |  |  |
| Daily Fruit servings | 1.03 (0.94,1.14) | 0.503 |  |  |
| Days/week vegetables | 1.09 (0.97,1.23) | 0.138 |  |  |
| Daily vegetable servings | 0.96 (0.82,1.13) | 0.624 |  |  |
| Heart failure | 1.35 (0.66,2.79) | 0.413 |  |  |
| Stroke | 2.26 (0.97,5.25) | 0.058 | 2.21 (0.93,5.24) | 0.072 |
| BMI | 0.97 (0.94,1.01) | 0.151 |  |  |
| Waist Circumference | 0.99 (0.97,1.01) | 0.331 |  |  |
| Hypertension duration (yrs) | 1.02 (0.99,1.05) | 0.115 |  |  |
| ACE-Inhibitors use | 0.68 (0.43,1.07) | 0.095 | 0.76 (0.46,1.25) | 0.279 |
| ARB use | 1.82 (1.17,2.84) | 0.008 | 1.57 (0.97,2.56) | 0.068 |
| Beta-Blockers use | 1.17 (0.63,2.18) | 0.611 |  |  |
| Ca Channel Blockers | 1.41 (0.72,2.74) | 0.316 |  |  |
| Diuretics | 1.41 (0.92,2.16) | 0.111 |  |  |
| Methyldopa | 0.72 (0.38,1.33) | 0.291 |  |  |
| Hydralazine | 1.42 (0.16,12.84) | 0.755 |  |  |
| Statin | 0.94 (0.27,3.26) | 0.926 |  |  |
| Antiplatelet | 1.1 (0.45,2.71) | 0.831 |  |  |
| No. of Antihypertensive | 1.22 (0.95,1.57) | 0.113 |  |  |
| Hillbone score | 0.97 (0.91,1.04) | 0.422 |  |  |

*NHIS= National Health Insurance Scheme, BMI= Body Mass Index, ACE= Angiotensin Converting Enzyme, ARB= Angiotensin Receptor Blocker, Ca= calcium. P-value< 0.005 is significant*
